# In silico comparison of pharmacokinetic properties of three extended half-life factor IX concentrates

**DOI:** 10.1007/s00228-021-03111-2

**Published:** 2021-02-24

**Authors:** Tim Preijers, Laura Bukkems, Max van Spengler, Frank Leebeek, Marjon Cnossen, Ron Mathôt

**Affiliations:** 1 Hospital Pharmacy-Clinical Pharmacology, Amsterdam University Medical Center, Meibergdreef 9, P.O. Box 22660, 1100 DD Amsterdam, Netherlands; 2 Department of Hematology, Erasmus MC, University Medical Center Rotterdam, Rotterdam, Netherlands; 3 Department of Pediatric Hematology, Erasmus MC—Sophia Children’s Hospital, University Medical Center Rotterdam, Rotterdam, Netherlands

**Keywords:** Factor IX, Hemophilia B, Pharmacokinetics, Half-life, Comparative study

## Abstract

**Purpose:**

Pharmacokinetic (PK) differences between the extended half-life (EHL) factor IX (FIX) concentrates for hemophilia B exist, which may influence hemostatic efficacy of replacement therapy in patients. Therefore, we aimed to evaluate the PK properties of three EHL-FIX concentrates and compare them to a standard half-life (SHL) recombinant FIX (rFIX) concentrate.

**Methods:**

Activity-time profiles of PEGylated FIX (N9-GP), FIX linked with human albumin (rIX-FP), FIX coupled to human IgG1 Fc-domain (rFIXFc), and SHL rFIX were simulated for 10,000 patients during steady-state dosing of 40 IU/kg once weekly (EHL-FIX) and biweekly (rFIX) using published concentrate specific population PK models.

**Results:**

Half-lives were respectively 80, 104, and 82 h for N9-GP, rIX-FP, and rFIXFc versus 22 h for rFIX. Between the EHL concentrates, exposure was different with area under the curve (AUC) values of 78.5, 49.6, and 12.1 IU/h/mL and time above FIX target values of 0.10 IU/mL of 168, 168, and 36 h for N9-GP, rIX-FP, and rFIXFc, respectively. N9-GP produced the highest median in vivo recovery value (1.70 IU/dL per IU/kg) compared with 1.18, 1.00, and 1.05 IU/dL per IU/kg for rIX-FP, rFIXFc, and rFIX, respectively.

**Conclusions:**

When comparing EHL products, not only half-life but also exposure must be considered. In addition, variation in extravascular distribution of the FIX concentrates must be taken into account. This study provides insight into the different PK properties of these concentrates and may aid in determination of dosing regimens of EHL-FIX concentrates in real-life.

## Introduction

Hemophilia B patients are characterized by a deficiency of coagulation factor IX (FIX) resulting in bleeding, typically in joints and muscles [1]. It has been demonstrated that patients with moderate and mild hemophilia—defined as a baseline FIX level of >0.01 IU/mL and >0.05 IU/mL, respectively—experience spontaneous bleeding less frequently and demonstrate delayed development of arthropathy when compared with severe hemophilia patients (<0.01 IU/mL) [2]. Therefore, traditionally severe hemophilia B patients administrate FIX concentrate prophylactically to maintain FIX trough levels of at least >0.01 IU/mL [3]. However, due to inter-individual variation in bleeding tendency the sufficient FIX target level during prophylaxis to prevent bleeding can vary between patients. Some patients do not experience bleeding when trough levels are <0.01 IU/mL while others require higher factor trough levels [4, 5]. In spite of these findings, it has been demonstrated in hemophilia A patients that longer time intervals spent with factor VIII activity levels >0.01 IU/mL resulted in lower annualized bleeding rates [6]. Some studies even suggested to aim for higher trough activity levels to prevent bleeds [7]. Therefore, higher FIX trough activity levels may be required for some patients, depending on bleeding tendency, level of physical activity, and joint status [8]. As a result, not only trough FIX activity levels but also area under the activity level versus time curve (AUC) and time spent with FIX activity levels above 0.03 IU/mL, 0.05 IU/mL, and 0.10 IU/mL are expected to be important determinants to predict bleeding risk.

Efforts have been made to modify the pharmacological properties of FIX concentrates in order to extend its terminal half-life and/or augment its in vivo hemostatic function [9, 10]. Currently, three extended half-life (EHL) FIX concentrates are widely available: PEGylated FIX (N9-GP), recombinant FIX linked with recombinant human albumin (rIX-FP), and FIX coupled to the human IgG1 Fc domain (rFIXFc) [11, 12]. Whereas standard half-life (SHL) FIX concentrates are generally administered twice weekly to maintain target FIX trough levels, EHL-FIX concentrates can be administered once weekly or possible even less frequently [13]. One of the greatest advantage of these EHL-FIX concentrates is the reduction in frequency of infusion, especially in patients with difficult venous access. On the contrary, less frequent administration of EHL-FIX concentrates may also result in longer time intervals at relatively low FIX activity levels, which may actually lead to lower hemostatic efficacy especially for patients requiring higher trough levels. For this reason, it is also important to examine the time patients spent above a specified FIX activity level.

Although the EHL-FIX products have been designed to have altered elongating PK properties when compared with SHL-rFIX products, these have not yet been simultaneously compared in a clinical study. A simultaneous comparison between PK properties of the EHL-FIX products can be useful, as the PK properties described in clinical trials are obtained with different dosing regimens making comparison of several PK properties difficult. Furthermore, in the reports of these clinical trials, clinically interesting PK properties such as time spent above a certain factor level are often not presented. Nevertheless, population PK models have been published for the examined concentrates, making evaluation using Monte Carlo simulations possible. Monte Carlo simulations not only allow the comparison of PK parameters in a typical or average patient but also illustrate the associated inter-patient variability observed in a patient population. Application of Monte Carlo simulations can be beneficial as costs and exposure of the patient to an intervention are minimized while maximizing similarity with clinical practice. Therefore, the objective of this study was to compare the PK properties of three currently available EHL-FIX concentrates to a widely used SHL-rFIX concentrate using Monte Carlo simulations.

## Methods

Monte Carlo simulations were performed to produce FIX activity levels versus time profiles of three EHL-FIX concentrates N9-GP (Refixia^®^, Novo Nordisk A/S, Denmark), rIX-FP (Idelvion^®^, CSL Behring GmbH, Germany), and rFIXFc (Alprolix^®^, Swedish Orphan Biovitrum AB, Sweden) and one SHL-rFIX concentrate (BeneFIX®, Pfizer, UK) in 10,000 virtual patients [14]. In a Monte Carlo simulation, a population PK model is used to generate individual PK parameters and subsequent FIX levels for each desired time-point. Residual error was not included in the simulated FIX levels. The simulations were performed with NONMEM v7.4.1. using population PK models reported in literature (Table 1) [15–17]. For N9-GP, only a population PK model based on phase 1 trial data was available in literature [18, 19]. In the phase 1 N9-GP trial, FIX levels were measured using a modified aPTT-based assay with a Trinity auto aPTT reagent (silica-based), while in the phase 3 trials, FIX levels were measured using an aPTT-based one-stage assay with a SynthAFax reagent [20, 21]. The activity of N9-GP is generally overestimated when a silica-based reagent is used, as applied in the phase 1 trial [22, 23]. Therefore, updated population PK parameters of N9-GP were generously provided by Novo Nordisk based on data from the phase 3 trials.

**Table 1:** Pharmacokinetic parameters of the population pharmacokinetic models used for simulation

Parameters	N9-GP^†,*^	rFIXFc^‡^ [15]	rIX-FP^§^ [16]	rFIX^§^ [17]
CL (mL/h)	0.5101	239	57	560
V1 (mL)	58.9213	7140	6480	6090
Q2 (mL/h)	-	167	29	22400
V2 (mL)	-	8700	1580	4160
Q3 (ml/h)	-	3930	-	430
V3 (ml)	-	3990	-	3900
Covariates				
Bodyweight effect on CL	-	0.436	0.53	0.66
Bodyweight effect on Q2 and Q3	-	-	-	0.66
Bodyweight effect on V1	-	0.396	0.79	0.64
Bodyweight effect on V2	-	-	0.79	0.64
Bodyweight effect on V3	-	-	-	0.64
Weight adjusted dose on V1	-	-	0.38	-
Age effect on V2 (% change with age different from 23 years)	-	-	-	1.6
Inter-individual variability (IIV)				
IIV on CL (%)	16.79^¶^	17.7	22.6	19.0
IIV on V1 (%)	14.06	21.7	26.9	46.0
IIV on Q2 (%)	-	35.8	-	-
IIV on V2 (%)	-	46.2	-	37.0
IIV on V3 (%)	-	37.7	-	28.0
Correlation between IIV Cl and V1 (%)	-	75.6	-	-
Inter-individual variability (IOV)				
IOV on CL (%)	-	15.1	-	-
IOV on V1 (%)	-	17.4	-	-
Residual variability				
Additive error (IU/ml)	0.01003	0.0024	0.0066	0.0064
Proportional error (%)	-	10.6	18.0	8.7

*CL* clearance, *V1* central volume of distribution, *Q2* inter-compartmental clearance of compartment 2, *V2* volume of compartment 2, *Q3* inter-compartmental clearance of compartment 3, *V3* volume of compartment 3

*Population pharmacokinetic parameters of N9-GP were provided by Novo Nordisk (personal communication)

^†^Parameters scaled to 1 kg

^**‡**^Parameters CL and V1 scaled to 73 kg by allometric scaling

^**§**^Parameters scaled to 70 kg by allometric scaling

^¶^IIV of clearance of N9-GP was taken from Collins [18]

R software (v3.4.3) was used to create the population of 10,000 virtual severe hemophilia B patients [24].

Different age and bodyweight characteristics were assigned to the virtual patients. The ranges of these simulated characteristics were based on the combined age and bodyweight ranges from the studied populations of the population PK models available in literature to avoid extrapolation. Therefore, simulated age and bodyweight ranged from 21 to 65 years and from 57.3 to 90 kg, respectively. The relation between age and weight and distribution of these characteristics was simulated using the tmvtnorm package in R. For reasons of simplicity, PK of the EHL-FIX was only evaluated in severe hemophilia B patients (endogenous baseline level <0.01 IU/mL). Consequently, no endogenous baseline FIX level was simulated for the virtual patients. The population PK model for rIX-FP contained a structural parameter to describe the baseline FIX levels of hemophilia B patients. This parameter was, however, subsequently discarded during the Monte Carlo simulations, as baseline FIX levels were <0.01 IU/mL.

In the simulations, steady-state PK was present in all patients, receiving 40 IU/kg of EHL-FIX once weekly and 40 IU/kg SHL-rFIX twice-weekly. For each virtual patient, the following PK parameters were calculated: terminal elimination half-life, AUC (from 0 to 168 h), maximum FIX activity level, in vivo recovery, and FIX trough activity level. Moreover, the time below and above 0.01, 0.03, 0.05, and 0.10 IU/mL was calculated. Furthermore, individual PK parameters were used to calculate the dose of FIX concentrate needed to achieve a steady-state FIX trough activity level of 0.01, 0.03, 0.05, and 0.10 IU/mL.

## Results

**Fig. 1:**
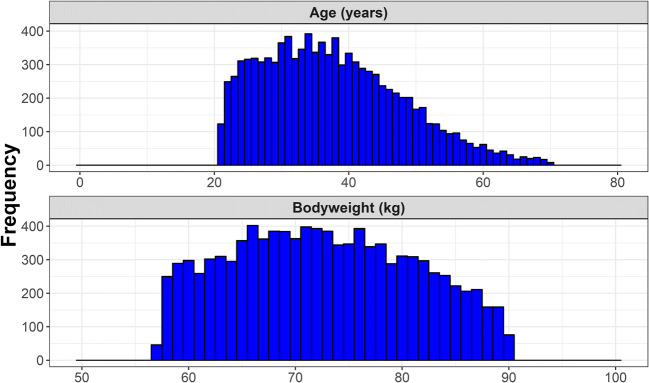
Distribution of age and bodyweight for the simulated population of 10,000 severe hemophilia B patients

**Fig. 2:**
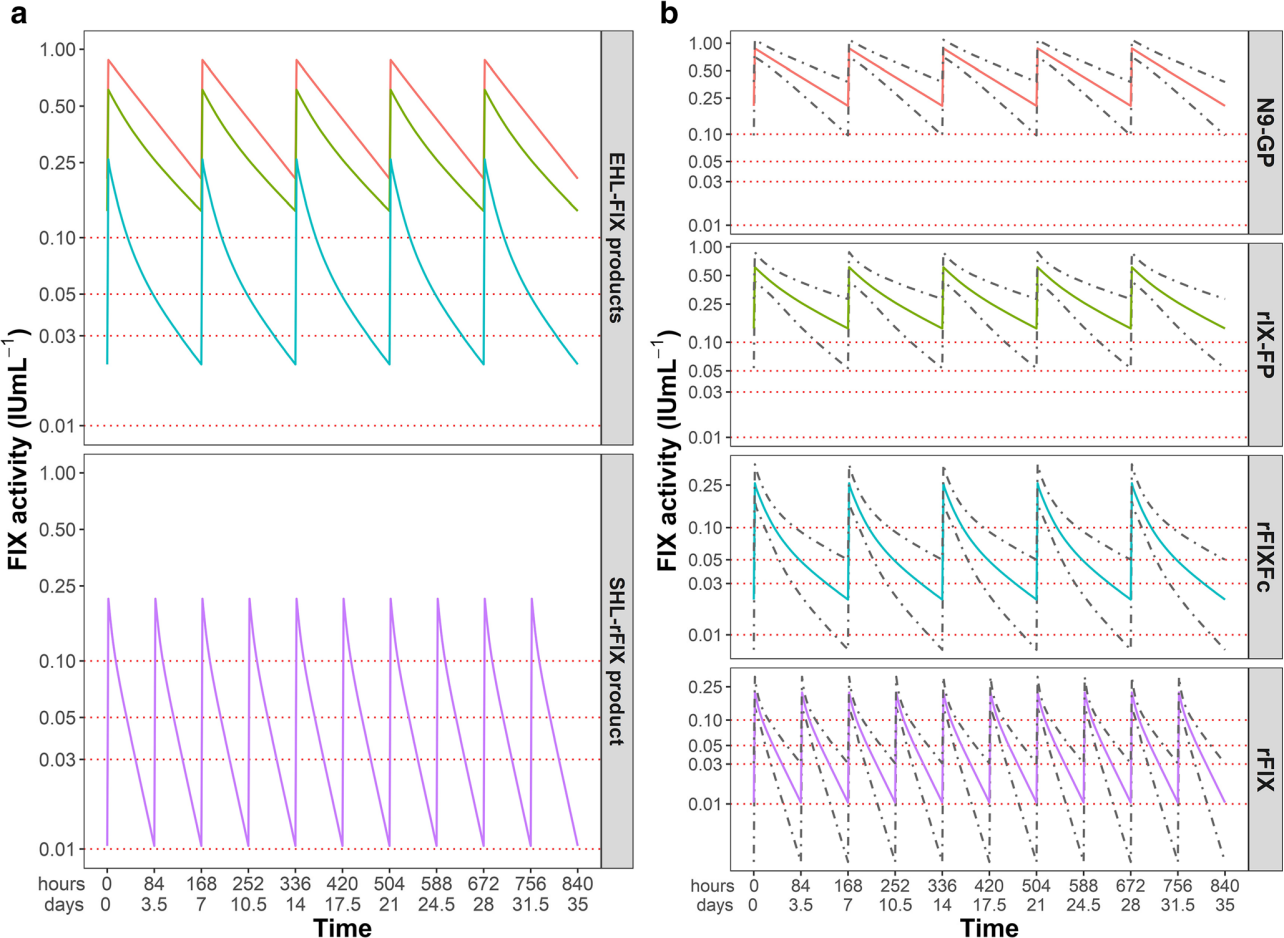
Simulated FIX activity levels for the examined FIX concentrates. IU, international units. SHL, standard half-life. EHL, extended half-life. **a** Median FIX activity levels versus time from N9-GP (orange), rIX-FP (green), rFIXFc (blue), and rFIX (purple) for 10,000 patients during steady-state dosing of 40 IU/kg once weekly (EHL-concentrates) and 40 IU/kg twice weekly (rFIX). The dashed red lines depict the FIX target trough levels. **b** Median simulated FIX activity levels from N9-GP (orange), rIX-FP (green), rFIXFc (blue), and rFIX (purple) versus time with the 2.5th and 97.5th percentiles (gray dashed lines) of the FIX activity levels. Note the logarithmically transformed *y*-axis

The distributions of age and bodyweight of the 10,000 virtual patients with severe hemophilia B are depicted in Figure 1. Figure 2 show that the FIX activity level versus time profiles vary between concentrates, demonstrating different PK properties such as exposure and half-life.

For rIX-FP, the longest elimination half-life was obtained (104 h), while the elimination half-lives of N9-GP and rFIXFc were comparable (80 and 82 h). As expected, these parameters were 4- to 5-fold longer than for the SHL-rFIX concentrate with a value of 22 h (Table 2). The increase in half-life of the various EHL-FIX concentrates did not result in comparable increases in exposure (AUC). The median AUC of N9-GP (78.5 IU/h/mL) was six times higher than the AUC of rFIXFc (12.1 IU/h/mL), while the AUC of rIX-FP was four times higher (49.6 IU/h/mL) than rFIXFc. This is also reflected in both the calculated trough FIX activity levels which are respectively 0.21, 0.14, and 0.02 IU/mL for N9-GP, rIX-FP, and rFIXFc, and in the time above and below 1, 3, 5, and 10 IU/mL (Table 2).

**Table 2:** Simulated pharmacokinetic parameters for steady-state dosing of 40 IU/kg

Parameter	N9-GP		rIX-FP		rFIXFc		rFIX	
	Median	Range 90%	Median	Range 90%	Median	Range 90%	Median	Range 90%
Terminal elimination half-life (h)	79.9	(56.0–115.1)	104.2	(73.6–158.8)	82.2	(47.5–158.9)	21.8	(14.1–34.5)
AUC (IU/h/mL)	78.5	(59.3–103.9)	49.6	(34.9–71.3)	12.1	(8.1–18.2)	10.1^†^	(7.27–14.0)
Maximum FIX activity level (IU/mL)	0.89	(0.74–1.08)	0.62	(0.46–0.86)	0.42	(0.27–0.66)	0.43	(0.23–0.80)
In vivo recovery (IU/dL per IU/kg)	1.70	(1.35–2.15)	1.18	(0.78–1.83)	1.00	(0.62–1.58)	1.05	(0.54–1.99)
Trough FIX activity level (IU/mL)	0.21	(0.11–0.35)	0.14	(0.06–0.26)	0.021	(0.009–0.045)	0.010	(0.002–0.027)
Time above 0.01 IU/mL (h)	168.0	(168.0–168.0)	168.0	(168.0–168.0)	168.0	(156.1–168.0)	168.0^†^	(112.2–168.0)
Time above 0.03 IU/mL (h)	168.0	[168.0–168.0)	168.0	(168.0–168.0)	129.6	(74.5–168)	100.1^†^	(65.9–157.3)
Time above 0.05 IU/mL (h)	168.0	(168.0–168.0)	168.0	(168.0–168.0)	80.8	(48.0–149.5)	68.8^†^	(44.8–108.5)
Time above 0.10 IU/mL (h)	168.0	(168.0–168.0)	168.0	(118.7–168.0)	36.1	(21.1–64.6)	30.4^†^	(19.7–48.6)
Dose to achieve target activity								
Target trough 0.01 IU/mL (IU/kg)	1.93	(1.16–3.68)	2.88	(1.55–6.20)	18.9	(9.0–46.0)	78.7^†^	(29.7–33.7)
Target trough 0.03 IU/mL (IU/kg)	5.80	(3.48–11.0)	8.63	(4.66–18.6)	56.7	(26.9–138.0)	236.1^†^	(89.0–911.2 )
Target trough 0.05 IU/mL (IU/kg)	9.66	(5.80–18.4)	14.4	(7.76–31.0)	94.6	(44.9–229.8)	393.6^†^	(148.3–1518)
Target trough 0.10 IU/mL (IU/kg)	19.32	(11.6–36.8)	28.8	(15.5–62.0)	189.1	(89.8–459.7)	787.1^†^	(296.7–3037)

The steady-state FIX activity levels of the EHL-FIX concentrates were achieved by dosing 40 IU/kg every 168 h, whereas steady-state FIX activity levels for rFIX were achieved by dosing 40 IU/kg every 84 h

*IU* international units, *AUC* area under the curve

^†^As rFIX doses were administrated twice weekly; the calculated value depicts the sum of the two doses administered per week

Although a weekly dose of 40 IU/kg produces median FIX activity levels above 0.01 IU/mL during the complete dosing period of 168 h (1 week) for each of the EHL-FIX concentrate, significant differences were observed for a target trough activity level of 0.10 IU/mL. In the latter case, median values for the time above a target activity level of 10 IU/mL were respectively 168, 168, and 36 h for N9-GP, rIX-FP, and rFIXFc. Interestingly, once weekly dosing of 40 IU/kg rFIXFc produced similar values for AUC and time above 10 IU/mL as compared with dosing of rFIX twice weekly. In Table 2, doses to maintain specific target trough activities levels are presented. In comparison with rFIX, the required weekly dose for a target trough activity level of 0.01 IU/mL was 40-, 27-, and 4.1-fold lower for N9-GP, rIX-FP, and rFIXFc, respectively. In our study, simulated trough activity level of the EHL-FIX concentrates at 168 h were in agreement with those clinically observed and reported in literature [15, 16, 18, 25].

In general, after administration of 40 IU/kg, higher peak FIX activity levels were observed for N9-GP and rIX-FP in comparison with rFIXFc. This is also reflected in the calculated in vivo recovery (IVR) values, with N9-GP showing the highest median IVR of 1.70 IU/dL per IU/kg. rIX-FP, rFIXFc, and rFIX produced lower median IVR values of 1.18, 1.00, and 1.05 IU/dL per IU/kg, respectively.

## Discussion

Using Monte Carlo simulations, individual PK parameters and subsequent FIX activity levels over time curves were obtained. The observed terminal half-life values of the EHL-FIX concentrates were comparable, with rIX-FP showing a slightly longer terminal half-life. On the other hand, N9-GP and rIX-FP clearly demonstrated higher exposure, higher trough FIX activity levels, longer time above a target level (0.03, 0.05, or 0.10 IU/mL) than rFIXFc. These results are comparable to the PK comparison between N9-GP and rFIX-Fc performed by Escuriola Ettingshausen et al. demonstrating favorable PK for N9-GP [26].

The lower exposure and shorter time above a certain target level of rFIXFc compared with the other EHL-FIX concentrates could indicate that higher rFIXFc doses or shorter dosing intervals are necessary with this concentrate especially for patients that require higher FIX trough levels or patients that require higher FIX activity levels for physical activities. However, it must be taken into account that the characteristic FIX activity level versus time profile of rFIXFc—with a rapid decreasing FIX activity level during the distribution phase and a slower decrease during the elimination phase—is possibly a result of extravascular FIX binding with collagen IV [27]. Just as for rFIX, rFIXFc distribution is not limited to the plasma, and the PK curve displays a rapid distribution to the extravascular compartment [15, 26]. In comparison, studies have observed that N9-GP mostly remains in plasma compartment, as the PEG moiety of N9-GP possibly reduces distribution to extravascular space [21, 28]. These differences in distribution are also illustrated by the fact that rFIX and rFIXFc are both described by three compartment models, while the PK of N9-GP and rIX-FP are described by one and two compartment models [15–17]. Several non-clinical studies have indicated that extravascular FIX plays a clinically relevant role in hemostasis, but the full extent of this pharmacodynamic effect is yet to be discovered [27, 29, 30]. Although annual bleeding rates (ABR) are not directly comparable, similar median and interquartile ranges of ABR were observed in clinical studies for rFIXFc in adult hemophilia B patients after weekly prophylaxis with similar doses (2.3, IQR 0.44–3.76; median dose 49.5 IU/kg) compared with rIX-FP (1.58, IQR: 0.00–4.06; median dose 40.3 IU/kg) and N9-GP (1.04, IQR 0.00–4.00; median dose 40 IU/kg) [31–33]. This may indicate that the hemostatic efficacy of rFIXFc is more or less similar despite lower FIX activity levels. As a result, the pharmacodynamic properties (“intrinsic efficacy”) of rFIXFc may be different from N9-GP and rIX-FP.

Since this study was performed in silico and used the published population PK models, the results can only be interpreted for a study population similar to the population on which the PK models were originally built. Therefore, the presented results reflect PK parameters for patients from 21 to 65 years and from 57.3 to 90 kg. Furthermore, the blood sampling schemes used for data collection of the population PK models may have influenced the PK properties, as prolonged FIX sampling increases the obtained terminal half-life [34, 35]. Additionally, it is important to realize that varying one-stage assays with varying activators have been applied in population PK studies performed by pharmaceutical companies and in clinical reports, which may additionally contribute to the found differences. Finally, it is important that the presented study results are based on simulations and should be interpreted with caution. Collection of real-world clinical data from patients is still essential, as for instance inter-patient (PK) variability may deviate in the clinical setting. Therefore, it is recommended to perform follow up clinical studies in which concentrates are compared using for instance a cross-over design.

## Conclusion

The simulations in this study show that PK properties of the novel EHL-FIX concentrates differ. Despite the comparable terminal half-lives that were obtained for the investigated EHL-FIX concentrates, different AUCs and different time intervals above a specific FIX activity level were obtained. This study gives insight into specific PK properties of the EHL-FIX concentrates and may therefore support FIX concentrate selection and determination of dosing regimens in the real-life setting of daily hemophilia care. However, to fully unravel the effect of the EHL-FIX concentrates on hemostatic efficacy in hemophilia B, further research exploring the dose and PK-pharmacodynamic relationship is warranted.

## Data Availability

For original data, please contact r.mathot@amsterdamumc.nl.

## Code availability

Please contact r.mathot@amsterdamumc.nl for code sharing.

## References

[1] Franchini M, Frattini F, Crestani S, Bonfanti C (2012). Haemophilia B: Current pharmacotherapy and future directions. Expert Opin Pharmacother.

[2] Ahlberg A (1965). Haemophilia in Sweden VII. Incidence, treatment and prophylaxis of arthropathy and other musculo-skeletal manifestations of haemophilia A and B. Acta Orthop Scand Suppl.

[3] Fijnvandraat K, Cnossen MH, Leebeek FWG, Peters M (2012). Diagnosis and management of haemophilia. BMJ.

[4] Ahnström J, Berntorp E, Lindvall K, Björkman S (2004). A 6-year follow-up of dosing, coagulation factor levels and bleedings in relation to joint status in the prophylactic treatment of haemophilia. Haemophilia.

[5] Bjorkman S (2003). Prophylactic dosing of factor VIII and factor IX from a clinical pharmacokinetic perspective. Haemophilia.

[6] Collins PW, Blanchette VS, Fischer K (2009). Break-through bleeding in relation to predicted factor VIII levels in patients receiving prophylactic treatment for severe hemophilia A. J Thromb Haemost.

[7] Den Uijl IEM, Fischer K, Van Der Bom JG (2011). Analysis of low frequency bleeding data: the association of joint bleeds according to baseline FVIII activity levels. Haemophilia.

[8] Iorio A, Iserman E, Blanchette V, Dolan G, Escuriola Ettingshausen C, Hermans C, Negrier C, Oldenburg J, Reininger A, Rodriguez-Merchan C, Spannagl M, Valentino LA, Young G, Steinitz-Trost KN, Gringeri A (2017). Target plasma factor levels for personalized treatment in haemophilia: a Delphi consensus statement. Haemophilia.

[9] Mancuso M, Santagostino E (2017). Outcome of clinical trials with new extended half-life FVIII/IX concentrates. J Clin Med.

[10] Graf L (2018). Extended half-life factor VIII and factor IX preparations. Transfus. Med. Hemotherapy.

[11] Taylor JA, Kruse-Jarres R (2016). A new era for hemophilia B treatment. Blood.

[12] Nazeef M, Sheehan JP (2016). New developments in the management of moderate-to-severe hemophilia B. J Blood Med.

[13] Collins P, Chalmers E, Chowdary P, Keeling D, Mathias M, O'Donnell J, Pasi KJ, Rangarajan S, Thomas A (2016). The use of enhanced half-life coagulation factor concentrates in routine clinical practice: guidance from UKHCDO. Haemophilia.

[14] Bonate PL (2001). A brief introduction to Monte Carlo simulation. Clin Pharmacokinet.

[15] Diao L, Li S, Ludden T, Gobburu J, Nestorov I, Jiang H (2014). Population pharmacokinetic modelling of recombinant factor IX Fc fusion protein (rFIXFc) in patients with haemophilia B. Clin Pharmacokinet.

[16] Zhang Y, Roberts J, Bensen-Kennedy D, Jacobs I, Santagostino E, Voigt C, Feussner A, Morfini M, Sidhu J (2016). Population pharmacokinetics of a new long-acting recombinant coagulation factor IX albumin fusion protein for patients with severe hemophilia B. J Thromb Haemost.

[17] Björkman S (2013). Population pharmacokinetics of recombinant factor IX: Implications for dose tailoring. Haemophilia.

[18] Collins PW, Møss J, Knobe K (2012). Population pharmacokinetic modeling for dose setting of nonacog beta pegol (N9-GP), a glycoPEGylated recombinant factor IX. J Thromb Haemost.

[19] Negrier C, Knobe K, Tiede A, Giangrande P, Møss J (2011). Enhanced pharmacokinetic properties of a glycoPEGylated recombinant factor IX: A first human dose trial in patients with hemophilia B. Blood.

[20] Oldenburg J, Carcao M, Lentz SR, Mahlangu J, Mancuso ME, Matsushita T, Négrier C, Clausen WHO, Ehrenforth S, Young G (2018). Once-weekly prophylaxis with 40 IU/kg nonacog beta pegol (N9-GP) achieves trough levels of >15% in patients with haemophilia B: Pooled data from the paradigm^TM^ trials. Haemophilia.

[21] Tiede A, Abdul-Karim F, Carcao M, Persson P, Clausen WHO, Kearney S, Matsushita T, Negrier C, Oldenburg J, Santagostino E, Young G (2017). Pharmacokinetics of a novel extended half-life glycoPEGylated factor IX, nonacog beta pegol (N9-GP) in previously treated patients with haemophilia B: results from two phase 3 clinical trials. Haemophilia.

[22] Rosén P, Rosén S, Ezban M, Persson E (2016). Overestimation of N-glycoPEGylated factor IX activity in a one-stage factor IX clotting assay owing to silica-mediated premature conversion to activated factor IX. J Thromb Haemost.

[23] Bowyer AE, Hillarp A, Ezban M, Persson P, Kitchen S (2016). Measuring factor IX activity of nonacog beta pegol with commercially available one-stage clotting and chromogenic assay kits: a two-center study. J Thromb Haemost.

[24] Core Team R (2018). R: A language and environment for statistical computing.

[25] Young G, Collins PW, Colberg T, Chuansumrit A, Hanabusa H, Lentz SR, Mahlangu J, Mauser-Bunschoten EP, Négrier C, Oldenburg J, Patiroglu T, Santagostino E, Tehranchi R, Zak M, Karim FA (2016). Nonacog beta pegol (N9-GP) in haemophilia B: a multinational phase III safety and efficacy extension trial (paradigm^TM^4). Thromb Res.

[26] Escuriola Ettingshausen C, Hegemann I, Simpson ML, Cuker A, Kulkarni R, Pruthi RK, Garly ML, Meldgaard RM, Persson P, Klamroth R (2019). Favorable pharmacokinetics in hemophilia B for nonacog beta pegol versus recombinant factor IX-Fc fusion protein: a randomized trial. Res Pract Thromb Haemost.

[27] Stafford DW (2016). Extravascular FIX and coagulation. Thromb J.

[28] Salas J, van der Flier A, Hong V (2017). Extravascular distribution of conventional and Ehl FIX products using in vivo SPECT imaging analysis in hemophilia B mice. Blood.

[29] Feng D, Stafford KA, Broze GJ, Stafford DW (2013). Evidence of clinically significant extravascular stores of factor IX. J Thromb Haemost.

[30] Cooley B, Funkhouser W, Monroe D, Ezzell A, Mann DM, Lin FC, Monahan PE, Stafford DW (2016). Prophylactic efficacy of BeneFIX vs Alprolix in hemophilia B mice. Blood.

[31] Pasi KJ, Fischer K, Ragni M, Nolan B, Perry DJ, Kulkarni R, Ozelo M, Mahlangu J, Shapiro AD, Baker RI, Bennett CM, Barnes C, Oldenburg J, Matsushita T, Yuan H, Ramirez-Santiago A, Pierce GF, Allen G, Mei B (2017). Long-term safety and efficacy of extended-interval prophylaxis with recombinant factor IX Fc fusion protein (rFIXFc) in subjects with haemophilia B. Thromb Haemost.

[32] Santagostino E, Martinowitz U, Lissitchkov T (2016). Long-acting recombinant coagulation factor IX albumin fusion protein (rIX-FP) in hemophilia B: results of a phase 3 trial. Blood.

[33] Collins PW, Young G, Knobe K, Karim FA, Angchaisuksiri P, Banner C, Gürsel T, Mahlangu J, Matsushita T, Mauser-Bunschoten EP, Oldenburg J, Walsh CE, Negrier C, paradigm 2 Investigators (2014). Recombinant long-acting glycoPEGylated factor IX in hemophilia B: a multinational randomized phase 3 trial. Blood.

[34] Hua B, Wu R, Sun FF, Luo B, Alvey C, LaBadie RR, Qu PR, Korth-Bradley JM, Rendo P (2017). Confirmation of longer FIX activity half-life with prolonged sample collection after single doses of nonacog alfa in patients with haemophilia B. Thromb Haemost.

[35] Mahlangu J, Powell JS, Ragni MV, Chowdary P, Josephson NC, Pabinger I, Hanabusa H, Gupta N, Kulkarni R, Fogarty P, Perry D, Shapiro A, Pasi KJ, Apte S, Nestorov I, Jiang H, Li S, Neelakantan S, Cristiano LM, Goyal J, Sommer JM, Dumont JA, Dodd N, Nugent K, Vigliani G, Luk A, Brennan A, Pierce GF, A-LONG Investigators (2014). Phase 3 study of recombinant factor VIII Fc fusion protein in severe hemophilia A. Blood.

